# The Royal Society

**Published:** 1903-12-05

**Authors:** 


### The Royal Society.

On St. Andrew's Day the Fellows of the Royal Society met to celebrate the anniversary of the Society's foundation, and to appoint officers for the ensuing year. In contrast to the peace and calm which usually characterise the business proceedings on these occasions, there was, last Monday, the excitement of a contested election. This was for the secretaryship rendered vacant by the retirement from office of Sir Michael Foster. Under ordinary circumstances the officers are nominated by the President and Council and accepted as a matter of course by the general body of Fellows. On this occasion it appears that the choice of the President and Council was not accepted in the customary manner by one section of the Fellows. Sir Archibald Geikie had been nominated to fill the vacant post. A certain number of the Fellows, however, supported the candidature of Professor Halliburton on the ground that it was highly desirable to adhere to the time-honoured usage of electing to one of the two secretaryships a Fellow whose work is completely biological in character, and one in active pursuit of such work. The result of the election was that Sir Archibald Geikie was chosen to fill the vacant post.

